# GMP-grade α-TEA lysine salt: a 28-Day oral toxicity and toxicokinetic study with a 28-Day recovery period in Beagle dogs

**DOI:** 10.1186/s12885-016-2220-6

**Published:** 2016-03-08

**Authors:** Bella S. Guerrouahen, Tobias Hahn, Zefora Alderman, Brendan Curti, Walter Urba, Emmanuel T. Akporiaye

**Affiliations:** Sidra Medical and Research Center, Experimental Biology Division – Research, PO Box 26999, Doha, Qatar; Laboratory of Tumor Immunology and Therapeutics, Earle A. Chiles Research Institute, Robert W. Franz Cancer Research Center, Providence Portland Medical Center, 4805 NE Glisan St. 2N35 Portland, OR USA

**Keywords:** Cancer therapy, Vitamin analog, α-TEA lysine salt, Toxicokinetic, Pharmacokinetics, Non-rodent

## Abstract

**Background:**

Alpha-tocopheryloxyacetic acid (α-TEA) is a semi-synthetic derivative of naturally occurring vitamin E (alpha-tocopherol) that can be delivered via an oral route. Preclinical in vitro and in vivo data demonstrated that α-TEA is a potent anti-tumor agent with a safe toxicity profile in mice. We report a comprehensive study to evaluate the toxokinetics of good manufacturing practice (GMP)-grade α-TEA in dogs after daily oral administration for 28 days, followed by a 28-day recovery period.

**Methods:**

Male and female beagle dogs received capsules of α-TEA Lysine Salt at doses of 100, 300, 1500 mg/kg/day. α-TEA plasma levels were determined by high-performance liquid chromatography (HPLC) with mass spectrometric detection. During the treatment, animals were observe for clinical signs, food consumption, body weight, and subjected to ophthalmoscopic, and electrocardiographic assessments. At the end of the dosing period, blood was taken and toxicokinetic analyses and histopathology assessments were performed when animals were necropsied.

**Results:**

Our findings showed that there was no α-TEA-related mortality or moribundity. At the highest dose, increases in white blood cells and fibrinogen levels were observed. These levels returned to normal at the end of the recovery period. Histopathological evaluation of major organs revealed no significant lesions related to α-TEA-treatment.

**Conclusion:**

We demonstrate that for designing clinical trials in patients, the highest non-severely toxic dose (HNSTD) of α-TEA is 1500 mg/kg/day in Beagle dogs and this data informed the design of dose-escalation studies of α-TEA in patients with advanced cancer.

## Background

Alpha-tocopheryloxyacetic acid (α-TEA) is a semi-synthetic, non-hydrolysable ether derivative of vitamin E. Structurally, α-TEA differs from vitamin E by the replacement of the hydroxyl group at the carbon number 6 of the phenolic ring of the chroman head with an acetic acid residue linked by an ether bound [1]. α-TEA is a cytotoxic drug that induces tumor cell death through targeting the mitochondria and by modulation of apoptosis and survival pathways [2]. The in vivo anti-tumor activity of α-TEA has been reported in several pre-clinical tumor models [1], and is partially dependent on a T-cell-mediated immune response [3–6]. Although α-TEA has been evaluated as an anti-cancer agent in numerous pre-clinical tumor models [1, 7–10], efforts to translate these findings into human clinical trials are lacking. The goal of this study was to conduct a dose-escalation evaluation in an appropriate non-rodent animal species as required by the United States Food and Drug Administration (U. S. FDA) to obtain relevant toxico- and pharmaco-kinetic information in preparation for a first-in-human trial to evaluate the safety and tolerability of α-TEA in patients with advanced cancer. α-TEA lysine salt was administered at increasing doses of 100, 300 and 1500 mg/kg to male and female beagle dogs for 28 consecutive days, and then observed for 28 days after the last α-TEA dose. Complete measurements of body weight and food consumption were conducted over the treatment period. Ophthalmologic and electrocardiographic observations were assessed and clinical pathology evaluated. In this dose escalation study utilizing the lysine salt of α-TEA, we demonstrated that daily administration of α-TEA was not toxic at 1500 mg/kg body weight. The findings from this comprehensive and observational study to evaluate the pharmacology and toxicology of the α-TEA (lysine salt) in beagle dogs formed the basis for initiating a first-in-human clinical trial in patients with advanced cancer that is ongoing (NCT 02192346).

## Methods

### Preparation of α –Tocopheryloxyacetic acid lysine salt in capsules

**Fig. 1:**
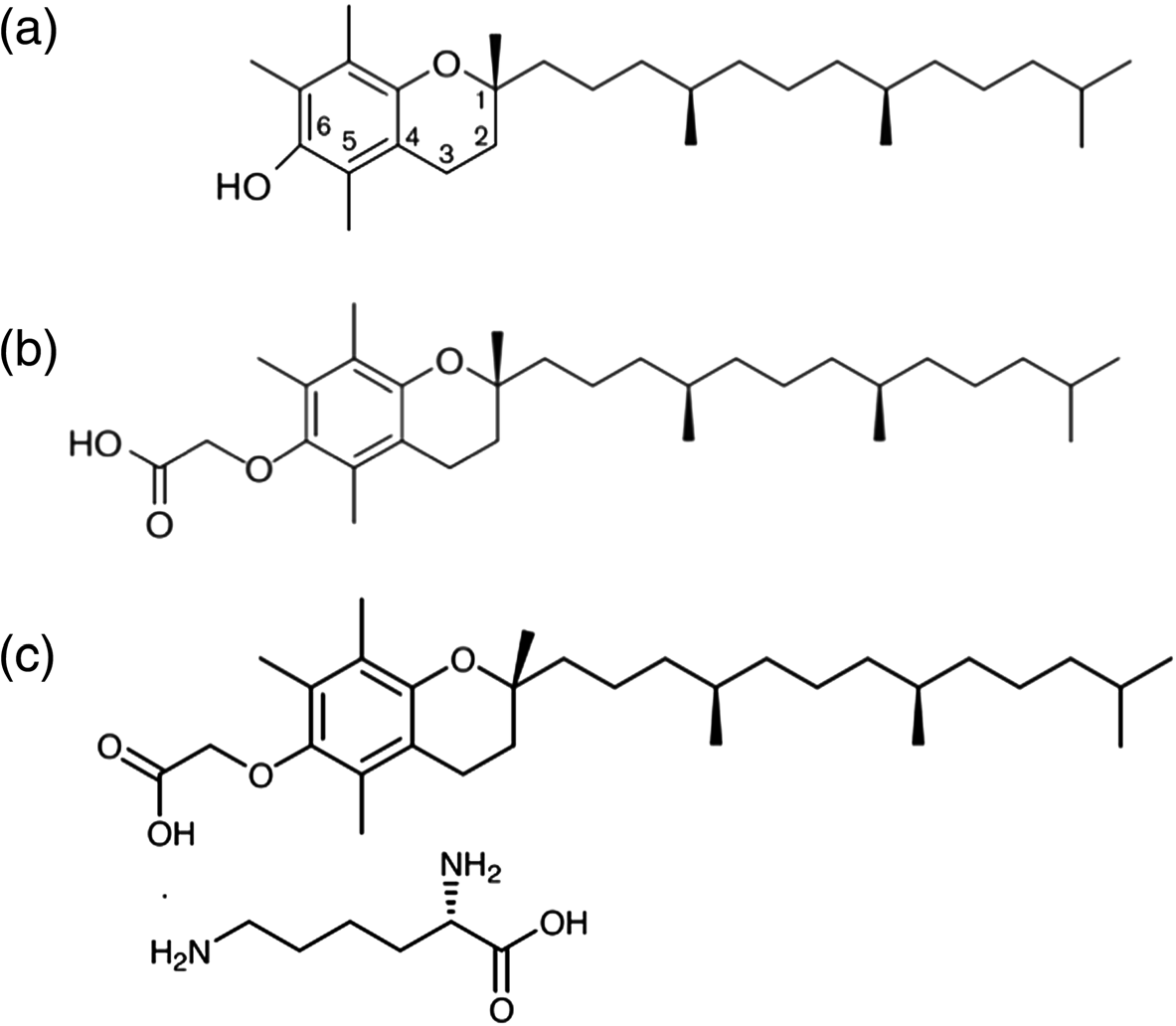
Chemical structure of α-Tocopherol, α-Tocopheryloxy Acetic Acid (α-TEA), and α-tocopheryloxy acetic acid Lysine Salt (α-TEA LS). **a** α-Tocopherol. Molecular Weight (MW) = 430.69. **b** α-Tocopheryloxy Acetic Acid (α-TEA). MW = 488. **c** α-TEA Lysine Salt. MW = 634.93

In the process of manufacturing α-TEA free acid for pre-clinical development, the contract Commercial Research Organization (Ricerca Biosciences, LLC, Concord OH) made the observation that during the initial scale-up, the free acid exhibited liquid crystal properties. Therefore, a salt screen was performed, which led to the identification and selection of the lysine salt. α-TEA lysine salt (α-TEA LS) was synthesized (Fig. 1), using a modification of a previously described procedure [9]. Briefly, α-TEA LS was prepared by reacting alpha-D-tocopherol with ethyl bromoacetate to form the ethyl ether intermediate. The ethyl ether intermediate was then reacted with potassium hydroxide to form α-TEA free acid. The lysine salt was formed by adding aqueous lysine solution to a solution of α-TEA in isopropyl alcohol. The lysine salt with its empirical formula of C_37_H_66_N_2_O_6_ and its molecular weight of 634.93 g/mol is a stable crystalline off-white powder. The dose concentration analysis was performed during the study and the test material was stable. Capsules for oral dose administration were prepared at least once weekly with appropriate amounts of bulk α-TEA placed into gelatin capsules (Pharmatek LLC, San Diego CA). Oral administration is the planned route of administration in humans. The dose of the drug is expressed as free acid using a correction factor of 1.3 to reflect salt content (calculated as ratio of lysine salt/free acid molecular weight).

### Animal studies

Initial studies in mice were conducted to assess the efficacy of the lysine salt form of α-TEA against established tumors. **Treatment of the animals (including but not limited to all husbandry, housing, and feeding conditions and euthanasia) was conducted in accordance with The Animal Welfare Act (Public Law 89-544) and the guidelines recommended in Guide for the Care and Use of Laboratory Animals (National Academy Press, Washington DC, 2011). The protocols and procedures involving the care and use of animals in the study were reviewed and approved by the Earle A. Chiles Research Institute and Ricerca Institutional Animal Care and Use Committees (IACUC).**

α-TEA was formulated in mouse chow and delivered to mice bearing established 4 T1 mammary tumors, as we previously described. This anti-tumor study was followed by a toxicology study. For this purpose, both the α-TEA free acid and α-TEA LS forms were administered to naïve mice intravenously or by oral gavage. A total of 44 Beagle dogs (*Canis familiaris*; were acquired by the contract CRO (Ricerca Biosciences) from Marshall BioResources, North Rose, NY) for the study. Animals were as uniform in age as possible; dogs were pre-pubertal to young adults, at least 8 months of age (range of 7.8 to 8.3 months of age) and weighed between 4 and 8 kg at the start of dose administration. The frequency of administration was designed to mimic the regimen intended for human trial. α-TEA LS was administered in gelatin capsules once daily to 22 male and 22 female dogs at 100, 300 and 1500 mg/kg for 28 consecutive days. The amount for each animal was based on the most recent body weight. Control animals received equal number of empty gelatin capsules as the high dose animals. Animals were observed for viability and clinical signs, daily food consumption, body weight (once weekly or twice for high dose animals). Group assignment and dose level are shown in the Table 1. Clinical pathology, ophthalmic and electrocardiography examinations were done at the pre-dose and after the terminal dose. Eight dogs from each group (4 females and 4 males) were necropsied at the end of the dosing period (Day 29) or at the end of the recovery period (Day 57). Animals were fasted overnight prior to scheduled blood test or necropsy. When necropsied, organs were collected and preserved in 10 % neutral-buffered formalin with the exception of the testes, epididymides, and eyes; which were fixed in Modified Davidson’s Solution for 24 to 48 h, water washed, and then transferred to 10 % neutral-buffered formalin for storage. Histopathology assessments were performed. Bone marrow smears were stained with Wright-Giemsa before cytology evaluation.

**Table 1:** Group assignment and dose levels

Group	Number of animals (M/F)	Test article	Nominal dose level (mg/kg/day)	Actual dose level^a^	Number of animals for necropsy (M/F)	
					Terminal (Day 29)	Recovery (Day 57)
1	6/6	Vehicle	0	0	4/4	2/2
2	4/4	α-TEA	100	130	4/4	-
3	6/6	α-TEA	300	390	4/4	2/2
4	6/6	α-TEA	1500	1950	4/4	2/2

^a^Dose levels have been corrected for lysine salt using a correction factor of 1.3

A total of 44 Beagle dogs (22 males and 22 females) received α-TEA lysine salt at different concentrations (0, 100, 300, 1500 mg/kg). Eight dogs from each group (4 females and 4 males) were necropsied at the end of dosing period (Day 29) or at the end of recovery period (Day 57)

### Toxicokinetics studies

Toxicokinetics studies were performed by Celerion Inc., a commercial research organization based in Lincoln, Nebraska, USA. Toxicokinetic parameters were studied on blood collected from the jugular vein of the animal (1 mL) on day 1 and on the last day of treatment. Blood samples were collected into K_3_EDTA-containing tubes at 1, 2, 4, 8, and 24 h following the first α-TEA administration. Following the last administration (day 28), blood collection for recovery animals was scheduled as follows: 2, 8, 24, 72, and 168 h. For the control animals (group 1), only the 8-hour sample was collected. The chosen time points were based on kinetic data gathered from pilot toxicity studies conducted in mice. Recovered plasma was stored at -70 °C until analysis.

### Data collection

Animal data, such as observations, body weights, food consumption, clinical pathology values, necropsy findings, and organ weights, were collected and reported electronically using Provantis™, Version 8 (Instem LSS Ltd. Staffordshire, UK). Urine was collected and analyzed by a Clinitek Atlas Urinalysis System. Data from the examination of urine sediment were entered directly into Provantis^TM^. Toxicokinetics data were collected and stored in electronic notebook system Labnotes^TM^ Client 1.18.

### Sample preparation and High Performance Liquid Chromatography (HPLC) analysis

Systemic levels of α-TEA were determined using HPLC with mass spectrometry. Bioanalytical data were obtained from Celerion, Inc according to the SOPs written based on the GLP principles. Briefly, an aliquot of the extracted dog plasma was analyzed by an HPLC equipped with an AB SCIEX API 4000^TM^ triple quadrupole mass spectrometer using an ESI source. Negative ions were monitored in the multiple reaction-monitoring (MRM) mode. Quantitation was determined using a weighted linear regression analysis (1/concentration^2^) of peak area ratios of the analyte and internal standard. The area under the plasma concentration-time curves, peak plasma concentration, time to achieve peak plasma concentration, and plasma terminal half-life (AUC, C_max_, T_max_, and T_1/2_) were determined using WinNonlin Version 6.2 (Pharsight, Mountain View CA), operating as a validated software system.

### Statistical analysis

For comparative statistics, data through the Day 29 termination were evaluated using the Levene Test for homogeneity of variances and the Shapiro-Wilks Test for normality of distributions, with significance at *p* ≤ 0.05. Data determined to be homogeneous and of normal distribution were evaluated for analysis of variance (ANOVA). If the ANOVA verified significance at *p* ≤ 0.05, pairwise comparisons of each treated group with the control group were made using a parametric test, Dunnett *t*-test, to identify statistical differences (*p* ≤ 0.05). Data determined to be nonhomogeneous or of non normal distribution were evaluated using a Kruskal-Wallis Test for group factor significance. If significance (*p* ≤ 0.05) existed between groups, a nonparametric test, Wilcoxon with Bonferroni-Holm, was used to compare treated groups to the control group.

## Results

### Anti-tumor activity of α-TEA lysine salt

**Fig. 2:**
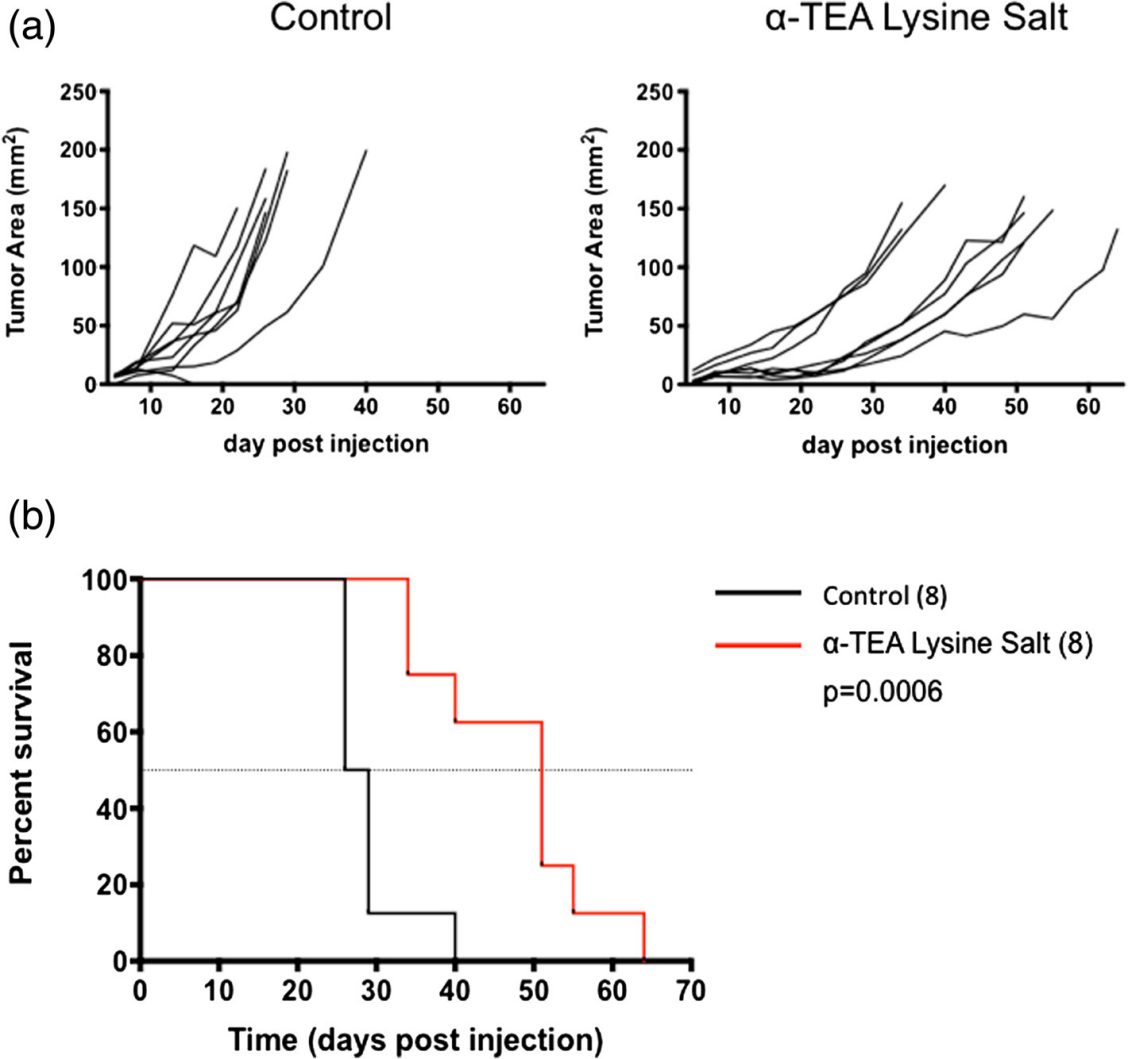
Anti-tumor activity of α-TEA lysine salt. **a** Effect of dietary delivery of α-TEA lysine salt on primary tumor growth. BALB/c mice were injected with 5x10^4^ 4 T1 tumor cells in the right mammary fat pad. When tumors became palpable (~5 days post tumor cell injection) mice received 390 mg/kg/day α-TEA lysine salt diet (equivalent to 300 mg/kg/day α-TEA) and tumor growth was monitored. **b** Oral α-TEA lysine salt prolongs survival of mammary tumor-bearing mice. BALB/c with established 4 T1 mammary tumors (day 5, ~ 4-6 mm^2^) received oral α-TEA lysine salt diet (390 mg/kg bodyweight) or nutrient-matched control diet. Mantel-Cox analysis of survival of n = 8 mice per group

**Fig. 3:**
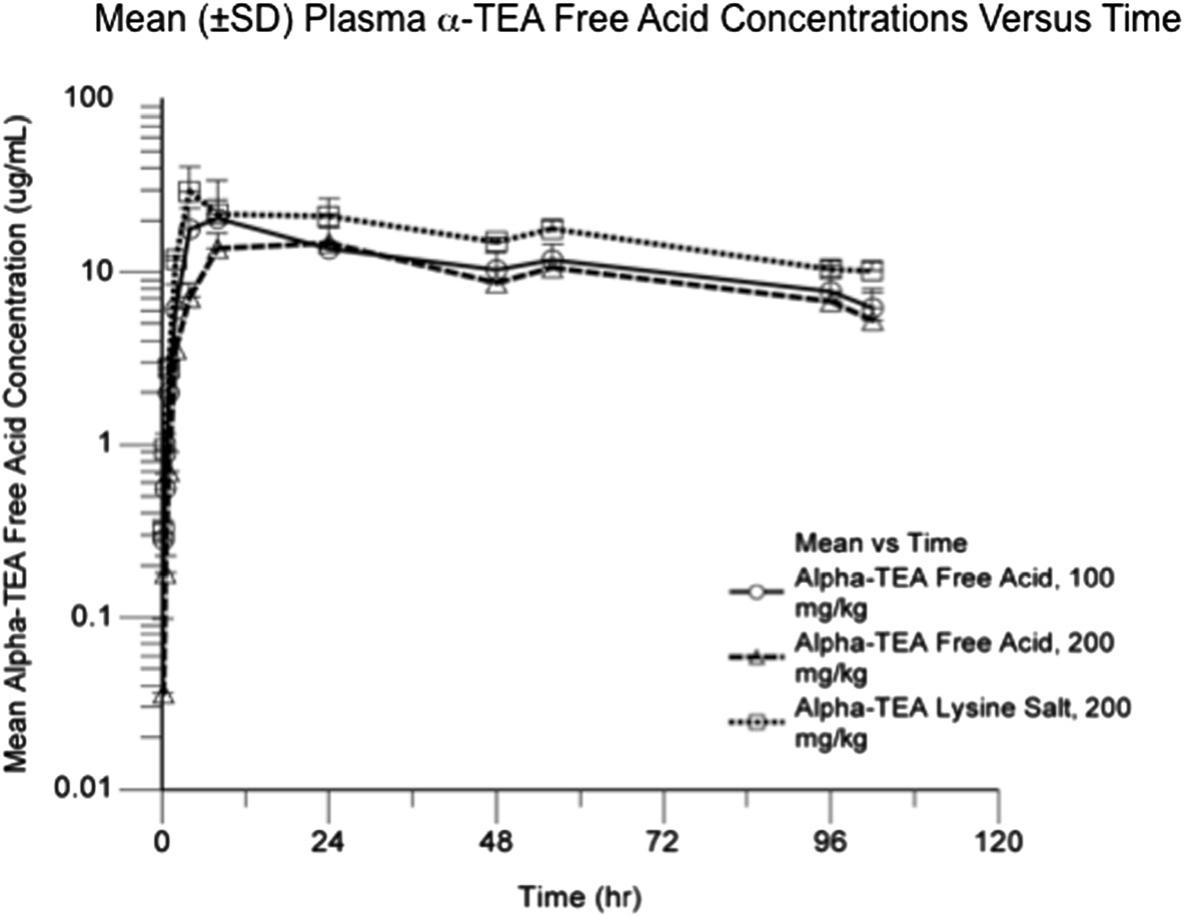
Exposure of BALB/c mice to a single-dose of lysine salt or free acid α-TEA. α-TEA free acid (100 mg/kg) and α-TEA lysine salt (200 mg/kg) were administered by intravenous injection or oral gavage. Blood was collected from 3 mice/group at each time point and analyzed for α-TEA levels by high performance liquid chromatography (HPLC) with mass spectrometric detection (LC-MS/MS)

We first tested the anti-tumor activity of GMP-manufactured α-TEA LS, by conducting experiments in BALB/c mice bearing established 4 T1 mammary tumors as previously described [11]. Tumor bearing mice received (i) control diet until 5 days post-tumor cell injection, and then were switched to 0.39 % α-TEA salt diet; or (ii) 390 mg/kg body weight α-TEA LS diet. After 60 days of tumor monitoring, we found that α-TEA LS significantly inhibit tumor growth (Fig. 2a) and prolonged overall survival compared to control diet (Fig. 2b). Blood samples were collected from 3 naïve mice/group and systemic levels of α-TEA were determined by HPLC with mass spectrometry detection in order to compare the pharmacokinetics and exposure of a single dose of α-TEA LS with that of the free acid. The free acid could only be administered by gavage because it was toxic when administered by intravenous injection. However, both routes of administration could be used with the lysine salt. As shown in Fig. 3 and Table 2, when α-TEA was administered orally by gavage at the same dose level (200 mg/kg), exposure was higher with α-TEA LS compared to α-TEA free acid. The peak plasma concentration after administration (C_max_) was 2 times higher with α-TEA LS compared to the α-TEA free acid (Fig. 3). We observed a saturation phenomenon reflected by a slight decrease in C_max_ following the increase in dose level from 100 mg/kg to 200 mg/kg of α-TEA free acid (Table 2). Our data indicate that the time to reach the peak plasma concentration after administration (T_max_) is 4 h with α-TEA LS versus 24 h for α-TEA free acid. The elimination half-lives was similar between 100 and 200 mg/kg α-TEA free acid and 200 mg/kg α-TEA lysine salt (54.6/50.2/54.6 h respectively).

**Table 2:** Effect of a single dose of α-TEA lysine salt or free acid on serum pharmacokinetics parameters

Test article	Dose level (mg/kg)	T_max_ (hr)	T_½_ (hr)	C_max_ (ug/mL)	AUC_0-24_ (hr*ug/mL)	AUC_0-∞_ (hr/*ug/mL)
α-TEA Free Acid	100	8	54.6	20.4	1,180	1,670
α-TEA Lysine Acid	200	4	54.	29.4	1,680	2,470
α-TEA Free Acid	200	24	50.2	14.7	1,020	1,400

α-TEA free acid (100 mg/kg) and α-TEA lysine salt (200 mg/kg) were administered by intravenous injection or oral gavage in BALB/c mice. Blood was collected from 3 mice/group at each time point and analyzed for α-TEA levels by HPLC with mass spectrometric detection (LC-MS/MS). The peak plasma concentration of a drug after administration (C_max_), the time required to reach it (T_max_), the time required for the concentration of the drug to reach half of its original value (T_1/2_), the area under the plasma drug concentration-time curve (AUC) were determined using WinNonlin Version 6.2 software system

### Repeat daily dosing of α-TEA Lysine Salt did not cause gross toxicity

**Fig. 4:**
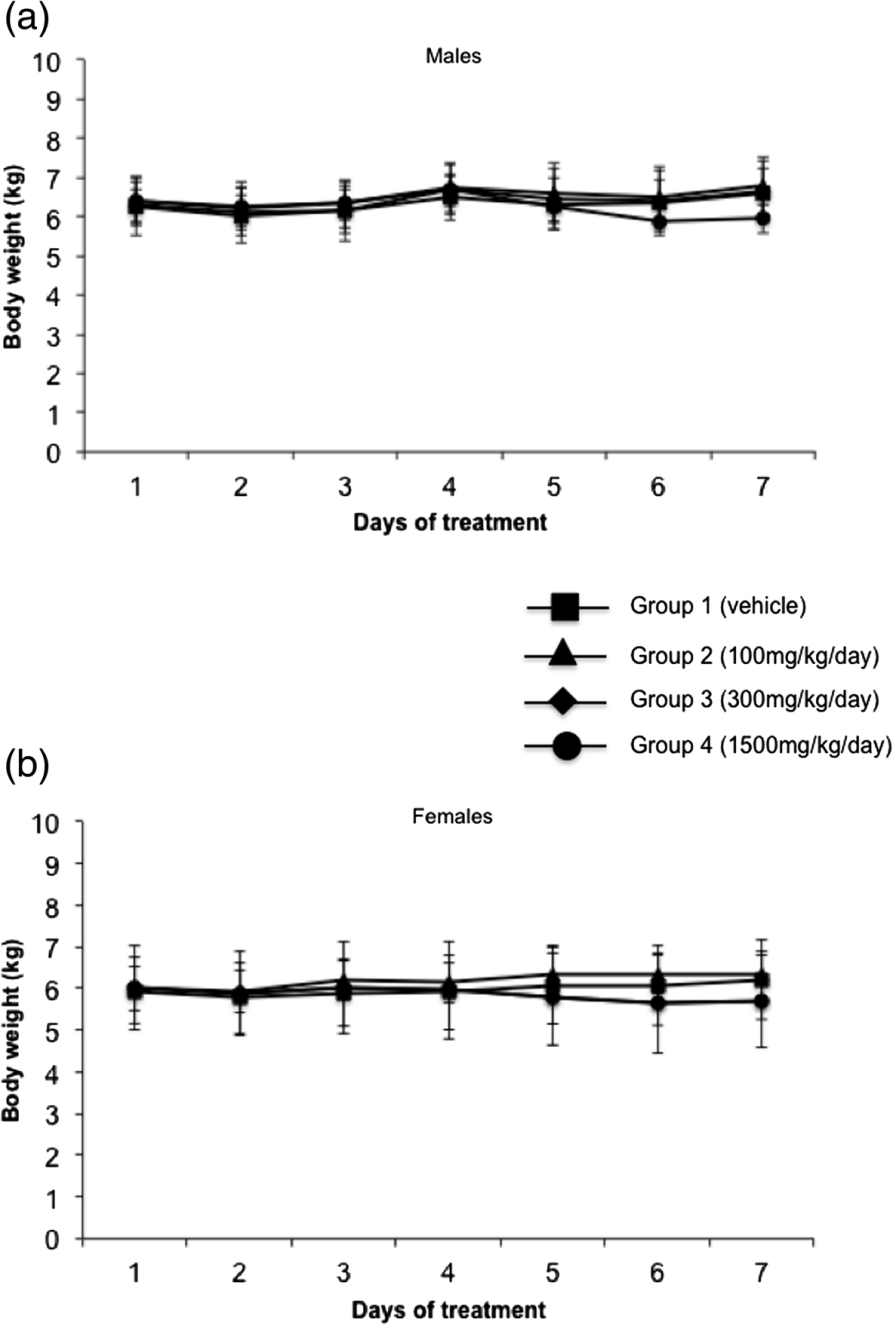
Measure of body weights during 28-day dosing of α-TEA lysine salt. Male (**a**) and female dogs (**b**) received indicated doses of α-TEA lysine salt daily. Body weights were determined twice before the start of the treatment and every week following the start of the treatment. Data points represent mean body weight ± SD

Several experiments were performed to evaluate the pharmacology and toxicology profile of repeated dosing of GMP-grade α-TEA LS in Beagle dogs, over a period of 28 days followed by a 28-day recovery period. Dose levels were chosen based on available data in mice [11] and from a preliminary dog tolerability study. Once daily, α-TEA salt was administered in gelatin capsules to male and female Beagle dogs at 100, 300 and 1500 mg/kg body weight for 28 consecutive days. Clinical signs were limited to fecal changes (decreased/discolored) and emesis in 1500 mg/kg animals on several occasions following start of dose administration and continuing until the start of recovery period. The decreased feces coincided with transient decreases in food consumption noted in several animals, and persisted upon continued dosing. α-TEA effects on food consumption at 1500 mg/kg were also reflected in the slightly decreased in body weight gain in all 1500 mg/kg males during the second and third week of dose administration and in two of six females during the second week and all the 1500 mg/kg females during the third week of administration (Fig. 4a, b). Individual body weight gain decreases were present at 300 and 100 mg/kg; however, overall group mean was comparable to controls and the changes were not considered toxicologically relevant. During the recovery period, the body weight gain in control and α-TEA-treated animals was comparable (data not shown). There was no moribundity or mortality throughout the course of treatment. There were no α-TEA-related ophthalmologic findings, but the 1500 mg/kg group showed sinus bradycardia on electrocardiograms at day 27 for the males and day 26 for the females.

### Clinical pathology (erythroid, leukocytes, coagulation parameters, serum chemistry and urinalysis) of α-TEA-treated animals

At the dosing level of 100, 300, and 1500 mg/kg, no effect on erythroid or serum chemistry was observed. On day 29, white blood cell counts (neutrophils and lymphocytes) and fibrinogen levels were slightly higher in animals that received 1500 mg/kg α-TEA LS (in 2 of 4 males and 2 of 4 females) compared to their pre-dose level (Tables 3 and 4). Interestingly, at the end of the recovery period, no α-TEA-related changes in coagulation or leukocyte parameters were recorded (Tables 5). On Day 29, evaluation of serum chemistry parameters showed that the mean chloride concentration (CL), mean blood urea nitrogen concentration (BUN), and mean aspartate aminotransferase activity (AST) values for the 1500 mg/kg males group were statistically higher compared to the controls. Although not statistically different, the mean CL, mean BUN, and mean AST values for the 1500 mg/kg females were minimally higher compared to the controls. The mean total protein concentration (TPRO), mean albumin (ALB) concentration, mean albumin to globulin ratio (A/G), and mean cholesterol (CHOL) concentration values for the 1500 mg/kg females were statistically lower compared to the controls. Although not shown to be statistically different, the mean glucose concentration (GLU) value for the 1500 mg/kg females group was lower compared to the controls (Tables 6 and 7). At Day 57 (Table 8), the mean BUN concentration value for the 300 mg/kg and 1500 mg/kg males groups was higher compared to the controls. This difference was attributed to the previous anorexia seen in the 1500 mg/kg males group, but was not of such a magnitude as to be considered an indication of renal toxicity. No urinalysis changes were reported at the highest dose (1500 mg/kg).

**Table 3:** Mean blood cells and coagulation parameters in dogs 10 days prior to treatment

		Vehicle			100 mg/kg			300 mg/kg			1500 mg/kg			Unit
		Mean	SD	N	Mean	SD	N	Mean	SD	N	Mean	SD	N	
Males	Erythrocytes													
	Red blood cells	6.315	0.485	6	6.418	0.316	4	6.523	0.211	6	6.74	0.416	6	M/ul
	Hemoglobin	13.98	1.13	6	13.83	0.71	4	14.43	0.48	6	14.8	1.21	6	g/L
	Hematocrit	43.18	3.51	6	43.08	1.54	4	44	1.5	6	45.23	3.2	6	%
	Mean corpuscular volume	63.38	0.97	6	67.18	1.55	4	67.52	2.46	6	67.1	1.42	6	fL
	Mean corpuscular hemoglobin	22.1	0.3	6	21.58	0.62	4	22.12	0.78	6	21.95	0.76		pg
	Mean corpuscular hemoglobin concentration	32.37	0.18	6	32.13	0.61	4	32.73	0.2	6	32.73	0.56	6	g/dL
	Reticulocytes	24.63	13.54	6	29.25	7.68	4	22.28	5.7	6	21.75	10	6	10^9^/L
	Leukocytes													
	White blood cells	7.473	1.869	6	7.373	1.858	4	9.175	2.951	6	7.103	1.64	6	K/uL
	Neutrophils	1.207	1.602	6	3.9	1.37	4	5.428	2.489	6	3.943	1.249	6	K/uL
	Lymphocytes	2.473	0.55	6	2.69	0.506	4	2.638	0.788	6	2.445	0.445	6	K/uL
	Monocytes	0.512	0.275	6	0.54	0.083	4	0.668	0.413	6	0.375	0.121	6	K/uL
	Eosinophils	0.128	0.077	6	0.14	0.022	4	0.282	0.171	6	0.193	0.108	6	K/uL
	Basophils	0.093	0.051	6	0.063	0.021	4	0.09	0.029	6	0.082	0.018	4	K/uL
	Thrombocytes													
	Platelets	381.7	39.4	6	330.8	80.1	4	427.8	82.2	6	360	57.4	6	K/uL
	Mean platelet volume	12.38	1.52	6	14.28	2.15	4	12.62	1.17	6	13.53	0.9	6	fL
	Protrombin time	6.63	0.24	6	6.88	0.17	4	6.8	0.3	6	6.85	0.19	6	Seconds
	Activated partial thromboplastin time	13.12	0.82	6	12.9	0.66	4	13.83	0.81	6	13.33	0.83	6	Seconds
	Fibrinogen	270.2	47.3	6	312.5	47.5	4	282	44.6	6	259.8	56.7	6	Mg/dL
Females	Erythrocytes													
	Red blood cells	6.562	0.322	6	6.788	0.297	4	6.49	0.417	6	6.59	0.344	6	M/ul
	Hemoglobin	14.62	0.8	6	15.43	0.77	4	14.12	1.52	6	14.7	0.79	6	g/L
	Hematocrit	45.15	2.42	6	46.98	2.33	4	43.78	3.88	6	45.22	2.26	6	%
	Mean corpuscular volume	68.82	2.46	6	69.2	1.83	4	67.4	2.3	6	68.63	1.52	6	fL
	Mean corpuscular volume hemoglobin	22.3	0.58	6	22.75	0.83	4	21.73	1.12	6	22.32	0.61	6	Pg
	Mean corpuscular hemoglobin concentration	32.4	0.41	6	32.85	0.31	4	32.17	0.65	6	32.55	0.21	6	g/dL
	Reticulocytes	32.4	15.63	6	41.9	19.22	4	34.42	14.98	6	25.3	11.84	6	10^9^/L
	Leukocytes													
	White blood cells	8.123	1.412	6	8.54	1.517	4	8.418	2.205	6	8.655	1.246	6	K/uL
	Neutrophils	4.873	1.151	6	4.855	1.139	4	4.88	1.404	6	5.457	0.986	6	K/uL
	Lymphocytes	2.482	0.62	6	2.793	0.381	4	2.578	0.459	6	2.482	0.213	6	K/uL
	Monocytes	0.473	0.079	6	0.513	0.131	4	0.505	0.185	6	0.353	0.119	6	K/uL
	Eosinophils	0.15	0.14	6	0.138	0.019	4	0.313	0.324	6	0.197	0.072	6	K/uL
	Basophils	0.093	0.047	6	0.148	0.038	4	0.08	0.029	6	0.102	0.014	6	K/uL
	Thrombocytes													
	platelets	358.8	83	6	350.5	81.3	4	319.2	53.1	6	356.7	49.3	6	K/uL
	Mean platelet volume	13.23	2.08	6	13.13	1.27	4	13.43	1.24	6	12.85	1.05	6	fL
	Prothrombin time	6.9	0.32	6	7.03	0.25	4	6.73	0.36	6	6.97	0.34	6	Seconds
	Activated partial thromboplastin time	13.42	0.65	6	13.4	0.41	4	13.37	0.44	6	13.12	0.37	6	Seconds
	Fibrinogen	227	54.8	6	225.8	90.2	4	219.2	42.6	6	205.8	35.3	6	mg/dL

Male and female dogs received indicated doses of α-TEA lysine salt daily for 28 days. Ten days before the treatment start, blood was collected from 6 dogs (0, 300, 1500 mg/kg treatment groups) or 4 dogs (100 mg/kg treatment group) and analyzed for blood cells and coagulation parameters

**Table 4:** Mean blood cells and coagulation parameters in dogs 29 days after the start date

		Vehicle			100 mg/kg			300 mg/kg			1500 mg/kg			Unit
		Mean	SD	N	Mean	SD	N	Mean	SD	N	Mean	SD	N	
Males	Erythrocytes													
	Red blood cells	6.238	0.841	4	6.438	0.598	4	6.21	0.143	4	6.418	0.437	4	M/ul
	Hemoglobin	14.55	1.91	4	14.55	1.1	4	6.21	0.143	4	6.418	0.437	4	g/L
	Hematocrit	42.1	6.09	4	42.18	3.27	4	41.05	1.04	4	41.7	2.79	4	%
	Mean corpuscular volume	67.45	1.1	4	65.63	1.51	4	66.08	2.16	4	65	1.75	4	fL
	Mean corpuscular hemoglobin	23.35	0.24	4	22.68	0.59	4	22.9	0.64	4	22.73	0.7	4	pg
	Mean corpuscular hemoglobin concentration	34.6	0.47	4	34.6	0.37	4	34.63	0.33	4	34.9	0.51	4	g/dL
	Reticulocytes	42.73	11.31	4	58.65	15.92	4	46	11.97	4	24.55	3.67	4	10^9^/L
	Leukocytes													
	White blood cells	7.438	0.919	4	9.23	1.25	4	12.23	3.277	4	11.91	3.574	4	K/uL
	Neutrophils	4.183	0.937	4	4.805	0.658	4	7.438	3.162	4	7.193	2.464	4	K/uL
	Lymphocytes	2.593	0.442	4	3.438	0.597	4	3.56	0.326	4	3.655	0.792	4	K/uL
	Monocytes	0.43	0.153	4	0.683	0.089	4	0.825	0.26	4	0.565	0.14	4	K/uL
	Eosinophils	0.095	0.039	4	0.178	0.055	4	0.223	0.101	4	0.388	0.328	4	K/uL
	Basophils	0.098	0.078	4	0.1	0.018	4	0.138	0.038	4	0.08	0.026	4	K/uL
	Thrombocytes													
	platelets	259.8	64	4	281.8	44.7	4	369.5	87.3	4	262.3	75.2	4	K/uL
	Mean platelet volume	10.95	1.11	4	9.88	0.8	4	9	0.87	4	8.6	0.77	4	fL
	Protrombin time	6.78	0.05	4	7.08	0.19	4	6.85	0.17	4	6.9	0.24	4	Seconds
	Activated partial thromboplastin time	12.68	1.16	4	12.7	0.84	4	13.5	0.88	4	13.05	1.28	4	Seconds
	Fibrinogen	254.5	42.7	4	288.5	18.9	4	289.5	85.4	4	414.5	89.9	4	mg/dL
Females	Erythrocytes													
	Red blood cells	6.33	0.241	4	6.895	0.563	4	6.72	0.306	4	6.773	0.383	4	M/ul
	Hemoglobin	14.85	1	4	16.43	1.7	4	15.55	1.1	4	15.78	0.8	4	g/L
	Hematocrit	43.33	3.14	4	47.68	4.73	4	45.48	3.24	4	45.98	2.05	4	%
	Mean corpuscular volume	68.33	2.53	4	69.1	1.14	4	67.83	3.07	4	67.88	1.6	4	fL
	Mean corpuscular volume hemoglobin	23.45	0.71	4	23.83	0.55	4	23.15	1	4	23.3	0.55	4	pg
	Mean corpuscular hemoglobin concentration	34.25	0.47	4	69.3	23.65	4	72.85	24.88	4	45.75	23.65	4	g/dL
	Reticulocytes	34.25	0.47	4	34.5	0.24	4	34.23	0.35	4	34.3	0.46	4	10^9^/L
	Leukocytes													
	White blood cells	8.81	3.586	4	9.75	2.4	4	9.743	1.237	4	13.31	1.721	4	K/uL
	Neutrophils	5.293	3.009	4	4.928	1.273	4	5.32	1.312	4	8.193	1.654	4	K/uL
	Lymphocytes	2.693	0.318	4	3.575	0.702	4	3.358	0.543	4	3.985	0.625	4	K/uL
	Monocytes	0.548	0.218	4	0.495	0.071	4	0.648	0.083	4	0.665	0.281	4	K/uL
	Eosinophils	0.163	0.111	4	0.505	0.467	4	0.273	0.1	4	0.3	0.124	4	K/uL
	Basophils	0.078	0.022	4	0.175	0.027	4	0.105	0.019	4	0.123	0.051	4	K/uL
	Thrombocytes													
	platelets	275.8	62.7	4	301.3	31.9	4	294.3	79	4	340	72.2	4	K/uL
	Mean platelet volume	13.03	2.23	4	10.45	0.79	4	10.85	0.97	4	9.75	0.71	4	fL
	Prothrombin time	7.1	0.18	4	7.18	0.15	4	6.8	0.22	4	6.93	0.19	4	Seconds
	Activated partial thromboplastin time	12.23	0.72	4	12.68	0.92	4	13.48	0.4	4	12.45	0.47	4	Seconds
	Fibrinogen	206	11.1	4	178.5	16.3	4	251.5	68.3	4	275.8	58.8	4	mg/dL

Male and female dogs received indicated doses of α-TEA lysine salt daily for 28 days. After 28 daily treatments, blood was collected from 4 dogs per treatment group and analyzed for blood cells and coagulation parameters

**Table 5:** Mean blood cells and coagulation parameters in dogs at the end of the recovery period (Day 57)

		Vehicle		300 mg/kg		1500 mg/kg		Unit
		Mean	N	Mean	N	Mean	N	
Males	Erythrocytes							
	Red blood cells	5.975	2	6.13	2	6.115	2	M/ul
	Hemoglobin	14	2	14.2	2	14.15	2	g/L
	Hematocrit	40	2	40.7	2	40.65	2	%
	Mean corpuscular volume	67	2	6.35	2	66.7	2	fL
	Mean corpuscular hemoglobin	23.4	2	23.15	2	23.2	2	pg
	Mean corpuscular hemoglobin concentration	34.9	2	34.85	2	34.8	2	g/dL
	Reticulocytes	34.15	2	78.3	2	59.92	2	10^9^/L
	Leukocytes							
	White blood cells	8.705	2	8.025	2	10.37	2	K/uL
	Neutrophils	5.275	2	4.53	2	6.14	2	K/uL
	Lymphocytes	2.7	2	2.645	2	3.055	2	K/uL
	Monocytes	0.34	2	0.225	2	0.385	2	K/uL
	Eosinophils	0.21	2	0.225	2	0.385	2	K/uL
	Basophils	0.125	2	0.125	2	0.125	2	K/uL
	Thrombocytes							
	platelets	229	2	290.5	2	310	2	K/uL
	Mean platelet volume	11.7	2	9.65	2	9.55	2	fL
	Protrombin time	7.05	2	7.15	2	7.15	2	Seconds
	Activated partial thromboplastin time	13.05	2	13.75	2	12.65	2	Seconds
	Fibrinogen							mg/dL
Females	Erythrocytes							
	Red blood cells	7.41	2	6.775	2	6.455	2	M/ul
	Hemoglobin	17.25	2	15.7	2	15.05	2	g/L
	Hematocrit	49.65	2	44.75	2	43.25	2	%
	Mean corpuscular volume	66.95	2	66	2	66.8	2	fL
	Mean corpuscular volume hemoglobin	23.3	2	23.15	2	23.3	2	pg
	Mean corpuscular hemoglobin concentration	34.8	2	35.1	2	34.85	2	g/dL
	Reticulocytes	69.4	2	68.25	2	52.3	2	10^9^/L
	Leukocytes							
	White blood cells	8.79	2	7.2	2	8.01	2	K/uL
	Neutrophils	4.925	2	3.895	2	4.915	2	K/uL
	Lymphocytes	2.87	2	2.785	2	2.475	2	K/uL
	Monocytes	0.34	2	0.215	2	0.275	2	K/uL
	Eosinophils	0445	2	0.16	2	0.2	2	K/uL
	Basophils	0.165	2	0.11	2	0.11	2	K/uL
	Thrombocytes							
	platelets	239.5	2	273	2	274.5	2	K/uL
	Mean platelet volume	11.3	2	11.2	2	10	2	fL
	Prothrombin time	6.7	2	7.35	2	6.8	2	Seconds
	Activated partial thromboplastin time	12.6	2	12.3	2	12.7	2	Seconds
	Fibrinogen	291	2	151.5	2	201.5	2	mg/dL

Male and female dogs received indicated doses of α-TEA lysine salt daily for 28 days. After 28 daily treatments, blood was collected from 2 dogs per treatment group and analyzed for blood cells and coagulation parameters

**Table 6:** Mean serum chemistry parameters in dogs 10 days prior to treatment

		Vehicle			100 mg/kg			300 mg/kg			1500 mg/kg			Unit
		Mean	SD	N	Mean	SD	N	Mean	SD	N	Mean	SD	N	
Males	Alanine transaminase (ALT)	58.2	47.5	6	55.8	42.2	4	37.7	5.4	6	36.8	15.2	6	U/L
	Aspartate transaminase (AST)	27.3	2.1	6	29.8	5.4	4	32.5	3.8	6	29.7	3.7	8	U/L
	Alkaline phosphatase (ALKP)	91.2	20.5	6	109.8	39.9	4	93.8	21.7	6	128.7	40.1	6	U/L
	Lactate deshydrogenase (LDH)	75.3	24.7	6	71.3	14.4	4	76	13.8	6	67.5	24.5	6	U/L
	Total bilirubin (TBILI)	0	0	6	0	0	4	0	0	0	0	0	6	mg/dL
	Gamma-Glutamyl transferase (GGT)	0	0	6	0	0	4	0	0	6	0	0	6	U/L
	Creatine phosphokinase (CK)	0	0	6	0	0	4	0	0	4	0	0	6	U/L
	Sodium (Na)	146	1.5	6	145.5	1	4	146.2	1.3	6	146.7	0.8	6	mmol/L
	Potassium (K)	4.65	0.19	6	4.65	0.17	4	113.3	1	6	113	1.1	6	mmol/L
	Chloride (CL)	113.5	1.5	6	113.3	1	4	113	1.1	6	113.5	2.4	6	mmol/L
	Calcium (CA)	10.13	0.19	6	10.28	0.15	4	10.43	0.3	6	10.53	0.45	0	mg/dL
	Inorganic phosphorus (PHOS)	5.43	0.77	6	5.98	0.7	4	5.23	0.38	6	5.3	0.45	6	mg/dL
	Blood urea nitrogen (BUN)	14.2	0.8	6	14.5	0.6	4	17	2.7	6	16	2.1	6	mg/dL
	Creatinine (CREA)	0.63	0.12	6	0.58	0.05	4	0.6	0	6	0.67	0.08	6	mg/dL
	Glucose (GLU)	97.3	3.5	5	91.3	8.3	4	92.3	7.4	6	96	8.9	6	mg/dL
	Total protein (TPRO)	4.75	0.24	6	4.98	0.29	4	4.9	0.14	6	4.87	0.26	6	g/dL
	Albumin (ALB)	2.88	0.17	6	2.93	0.26	4	2.92	0.12	6	2.83	0.28	6	g/dL
	Globulin (GLOB)	1.87	0.18	6	2.05	0.13	4	1.98	0.04	6	2.03	0.23	6	g/dL
	Albumin/Globulin ratio (A/G)	1.555	0.165	6	1.431	0.155	4	1.471	0.051	6	1.415	0.249	6	Ratio
	Cholesterol (CHOL)	168.3	48.5	6	160.8	9.3	4	150.5	31.5	6	163.8	24	6	mg/dL
	Triglycerides (TRIG)	37.3	8.2	6	38.3	5.9	4	36	4	6	40.2	5	6	mg/dL
Females	Alanine transaminase (ALT)	31.8	6.6	6	32.8	7.4	4	30.3	4.1	6	37.5	16.3	6	U/L
	Aspartate transaminase (AST)	29.3	4.1	6	31.8	11.2	4	27.5	3.4	6	28.3	3.7	6	U/L
	Alkaline phosphatase (ALKP)	100	32	6	83.8	18.3	4	95.7	25.5	6	75.3	13.7	6	U/L
	Lactate deshydrogenase (LDH)	60.3	18.1	6	65.3	25.1	4	52.2	15.4	6	62.2	21.8	6	U/L
	Total bilirubin (TBILI)	0.02	0.04	6	0	0	4	0	0	6	0	0	6	mg/dL
	Gamma-Glutamyl transferase (GGT)	0	0	6	0	0	4	0	0	6	0	0	6	U/L
	Creatine phosphokinase (CK)	165.7	37.4	6	161.5	73.7	4	141	30.1	6	142	43.6	6	U/L
	Sodium (Na)	146.8	1.5	6	147.3	1.7	4	146.7	1	6	147.3	0.8	6	mmol/L
	Potassium (K)	4.48	0.26	6	4.48	0.05	4	4.43	0.31	6	4.43	0.21	6	mmol/L
	Chloride (CL)	114.2	2.1	6	116	2.2	4	114.7	2.9	6	115	1.3	6	mmol/L
	Calcium (CA)	10.48	0.26	6	10.58	0.21	4	10.95	0.27	6	10.82	0.31	6	mg/dL
	Inorganic phosphorus (PHOS)	5.3	0.23	6	4.83	0.31	4	5.35	0.55	6	5.17	0.5	6	mg/dL
	Blood urea nitrogen (BUN)	15.2	2.3	6	13.3	1	4	14.2	2.6	6	14	3	6	mg/dL
	Creatinine (CREA)	0.57	0.05	6	0.6	0	4	0.58	0.08	6	0.65	0.08	6	mg/dL
	Glucose (GLU)	99.3	5	6	99.3	7.5	4	99.7	7.4	6	94.5	7	6	mg/dL
	Total protein (TPRO)	4.85	0.27	6	5	0.28	4	4.8	0.14	6	5.13	0.16	6	g/dL
	Albumin (ALB)	2.95	0.26	6	3.1	0.14	4	2.95	0.18	6	3.13	0.16	4	g/dL
	Globulin (GLOB)	1.9	0.09	6	1.9	0.18	4	1.85	0.14	6	2	0	6	g/dL
	Albumin/Globulin ratio (A/G)	1.556	0.157	6	162.3	10.5	4	1.607	0.21	6	1.567	0.082	6	Ratio
	Cholesterol (CHOL)	160	20.2	6	162.3	10.5	4	142.3	11.3	6	158.2	33	6	mg/dL
	Triglycerides (TRIG)	37.3	3.8	6	36	14.1	4	34	4.8	6	46.3	10.4	6	mg/dL

Male and female dogs received indicated doses of α-TEA lysine salt daily for 28 days. Ten days before the treatment start, blood was collected from 6 dogs per treatment group (0, 300, 1500 mg/kg) or 4 dogs (100 mg/mL) and analyzed for serum chemistry parameters

**Table 7:** Mean serum chemistry parameters 29 days after the start date in male dogs

		Vehicle			100 mg/kg			300 mg/kg			1500 mg/kg			Unit
		Mean	SD	N	Mean	SD	N	Mean	SD	N	Mean	SD	N	
Males	Alanine transaminase (ALT)	33.5	9.9	4	32	8.6	4	37.3	6.8	4	51	25.9	4	U/L
	Aspartate transaminase (AST)	34.8	3.2	4	36.5	5.7	4	46	8.8	4	49.8	5.9	4	U/L
	Alkaline phosphatase (ALKP)	96	24.4	4	104.3	37.1	4	116	71.9	4	227	315.9	4	U/L
	Lactate deshydrogenase (LDH)	57.8	6.4	4	61	18.7	4	70	21.8	4	55	18.9	4	U/L
	Total bilirubin (TBILI)	0.03	0.05	4	0.03	0.05	4	0.03	0.05	4	0	0	4	mg/dL
	Gamma-Glutamyl transferase (GGT)	0	0	4	0	0	4	0	0	4	0	0	4	U/L
	Creatine phosphokinase (CK)	246.3	79.8	4	185.3	61.2	4	272.3	145.6	4	133	29.1	4	U/L
	Sodium (Na)	148.5	1.3	4	148	0.8	4	147.8	0.5	4	148	1.4	4	mmol/L
	Potassium (K)	4.35	0.31	4	4.5	0.26	4	4.08	0.32	4	4.48	0.21	4	mmol/L
	Chloride (CL)	115.8	1	4	115	0.8	4	115	1.3	4	118.8	0.5	4	mmol/L
	Calcium (CA)	10.83	0.25	4	10.88	0.33	4	11.05	0.13	4	10.35	0.29	4	mg/dL
	Inorganic phosphorus (PHOS)	5.13	0.75	4	5.45	0.29	4	4.68	0.26	4	3.98	0.32	4	mg/dL
	Blood urea nitrogen (BUN)	18.3	2.1	4	18.8	3.3	4	19.3	1.9	4	24.3	1.7	4	mg/dL
	Creatinine (CREA)	0.6	0.14	4	0.6	0	4	0.58	0.1	4	0.55	0.08	4	mg/dL
	Glucose (GLU)	102.5	4	4	96.3	9	4	96.8	11.2	4	80.8	8.4	4	mg/dL
	Total protein (TPRO)	5.25	0.3	4	5.38	0.28	4	5.38	0.24	4	4.45	0.42	4	g/dL
	Albumin (ALB)	3.33	0.26	4	3.23	0.29	4	3.18	0.21	4	2.45	0.1	4	g/dL
	Globulin (GLOB)	1.93	0.1	4	2.15	0.06	4	2.2	0.14	4	2	0.34	4	g/dL
	Albumin/Globulin ratio (A/G)	1.729	0.141	4	1.502	0.152	4	1.448	0.14	4	1.247	0.184	4	Ratio
	Cholesterol (CHOL)	168.8	35.4	4	176	28	4	152.3	19.4	4	104.5	183.4	4	mg/dL
	Triglycerides (TRIG)	34	8.1	4	41.3	9.7	4	33.3	5.6	4	45.5	14.3	4	mg/dL
Females	Alanine transaminase (ALT)	27.3	5.1	4	26.3	1.9	4	35.8	10.2	4	45.5	20.9	4	U/L
	Aspartate transaminase (AST)	29	4.7	4	36.3	5.3	4	31.5	3.1	4	45.8	19.5	4	U/L
	Alkaline phosphatase (ALKP)	98.8	12.4	4	78.8	22.5	4	178	131.5	4	251.3	211.2	4	U/L
	Lactate deshydrogenase (LDH)	82.3	25.8	4	74	19.8	4	69.5	5.9	4	73	19.5	4	U/L
	Total bilirubin (TBILI)	0.03	0.05	4	0.05	0.06	4	0.03	0.05	4	0.03	0.05	4	mg/dL
	Gamma-Glutamyl transferase (GGT)	0	0	4	0.3	0.5	4	0	0	4	0	0	4	U/L
	Creatine phosphokinase (CK)	162.8	54.3	4	198.8	44.1	4	162.3	40.6	4	147.3	47.6	4	U/L
	Sodium (Na)	147.8	1	4	148.5	1.9	4	148.5	0.6	4	148.3	1	4	mmol/L
	Potassium (K)	4.4	0.34	4	4.53	0.17	4	4.38	0.31	4	4.65	0.34	4	mmol/L
	Chloride (CL)	114.5	1.7	4	115.5	1	4	115.5	1	4	117.8	2.8	4	mmol/L
	Calcium (CA)	10.7	0.35	4	11.05	0.38	4	11.05	0.37	4	10.25	0.84	4	mg/dL
	Inorganic phosphorus (PHOS)	5.03	0.54	4	4.25	0.9	4	4.4	0.47	4	4.35	0.58	4	mg/dL
	Blood urea nitrogen (BUN)	17.5	2.4	4	14.5	1.3	4	18.5	1.3	4	23.5	7.9	4	mg/dL
	Creatinine (CREA)	0.5	0.08	4	0.58	0.1	4	0.53	0.1	4	0.63	0.05	4	mg/dL
	Glucose (GLU)	103.3	4.8	4	102.8	11.5	4	108.8	7.8	4	84.8	2.1	4	mg/dL
	Total protein (TPRO)	5.28	0.17	4	5.35	0.31	4	5.3	0.22	4	4.65	0.3	4	g/dL
	Albumin (ALB)	3.35	0.17	4	3.38	0.25	4	3.35	0.19	4	2.8	0.22	4	g/dL
	Globulin (GLOB)	1.93	0.1	4	1.98	0.1	4	1.95	0.17	4	1.85	0.13	4	g/dL
	Albumin/Globulin ration (A/G)	1.745	0.142	4	1.71	0.109	4	1.729	0.191	4	1.516	0.118	4	Ratio
	Cholesterol (CHOL)	167.5	24.6	4	179	22.4	4	147.3	10.6	4	98.3	13	4	mg/dL
	Triglycerides (TRIG)	40	9.8	4	42	8.8	4	43.5	5.8	4	50	8.3	4	mg/dL

Male dogs received indicated doses of α-TEA lysine salt daily for 28 days. After 28 daily treatments, blood was collected from 4 dogs per treatment group and analyzed

**Table 8:** Mean serum chemistry parameters in dogs at the end of the recovery period (Day 57)

		Vehicle		300 mg/kg		1500 mg/kg		Unit
		Mean	N	Mean	N	Mean	N	
Males	Alanine transaminase (ALT)	33	2	36.5	2	33	2	U/L
	Aspartate transaminase (AST)	31	2	30.5	2	33	2	U/L
	Alkaline phosphatase (ALKP)	81.5	2	66	2	95	2	U/L
	Lactate deshydrogenase (LDH)	36	2	50	2	45.5	2	U/L
	Total bilirubin (TBILI)	0.05	2	0.05	2	0.1	2	mg/dl
	Gamma-Glutamyl transferase (GGT)	0	2	1	2	0	2	U/L
	Creatine phosphokinase (CK)	119	2	142.5	2	169	2	U/L
	Sodium (Na)	147.5	2	146.5	2	148.5	2	mmol/L
	Potassium (K)	4.3	2	4.6	2	4.4	2	mmol/L
	Chloride (CL)	116	2	115	2	116.5	2	mmol/L
	Calcium (CA)	10.65	2	10.45	2	10.55	2	mg/dL
	Inorganic phosphorus (PHOS)	5.15	2	5.1	2	5.3	2	mg/dL
	Blood urea nitrogen (BUN)	13.5	2	15.5	2	21	2	mg/dL
	Creatinine (CREA)	0.65	2	0.6	2	0.7	2	mg/dL
	Glucose (GLU)	99	2	99.5	2	96	2	mg/dL
	Total protein (TPRO)	5.3	2	5.15	2	5.4	2	g/dL
	Albumin (ALB)	3	2	2.9	2	2.95	2	g/dL
	Globulin (GLOB)	2.3	2	2.25	2	2.45	2	g/dL
	Albumin/Globulin ratio (A/G)	1.314	2	1.289	2	1.219	2	Ratio
	Cholesterol (CHOL)	149	2	154	2	163.5	2	mg/dL
	Triglycerides (TRIG)	24	2	24	2	41	2	mg/dL
Females	Alanine transaminase (ALT)	22.5	2	31	2	27.5	2	U/L
	Aspartate transaminase (AST)	33	2	29	2	26.5	2	U/L
	Alkaline phosphatase (ALKP)	73	2	63.5	2	50	2	U/L
	Lactate deshydrogenase (LDH)	52	2	49.5	2	47	2	U/L
	Total bilirubin (TBILI)	0.1	2	0.1	2	0.1	2	mg/dL
	Gamma-Glutamyl transferase (GGT)	0	2	0	2	0	0	U/L
	Creatine phosphokinase (CK)	154.5	2	146	2	120	2	U/L
	Sodium (Na)	146.5	2	146.5	2	147	2	mmol/L
	Potassium (K)	4.5	2	4.5	2	4.1	2	mmol/L
	Chloride (CL)	115.5	2	115.5	2	116.5	2	mmol/L
	Calcium (CA)	10.95	2	10.65	2	10.5	2	mg/dL
	Inorganic phosphorus (PHOS)	5.15	2	4.95	2	4.5	2	mg/dL
	Blood urea nitrogen (BUN)	15.5	2	13.5	2	14.5	2	mg/dL
	Creatinine (CREA)	0.65	2	0.7	2	0.65	2	mg/dL
	Glucose (GLU)	103.5	2	101.5	2	100	2	mg/dL
	Total protein (TPRO)	5.7	2	5.15	2	5.25	2	g/dL
	Albumin (ALB)	3.3	2	3	2	3.1	2	g/dL
	Globulin (GLOB)	2.4	2	2.15	2	2.15	2	g/dL
	Albumin/Globulin ratio (A/G)	1.377	2	1.395	2	1.444	2	Ratio
	Cholesterol (CHOL)	190.5	2	152	2	161	2	mg/dL
	Triglycerides (TRIG)	29.5	2	30.5	2	32	2	mg/dL

Male and female dogs received indicated doses of α-TEA lysine salt daily for 28 days. At the end of the recovery period, on day 57, blood was collected from 2 dogs per treatment group and analyzed

#### Histology of major organs

Dogs were euthanized the day following the end of dosing period or following a 28-day recovery period (day 57) by an overdose of sodium pentobarbital followed by exsanguination. A necropsy with tissue collection was conducted and included examination of the carcass and muscular/skeletal system, orifices, cranial cavity and external surface of the brain, neck, thoracic, abdominal and pelvic cavities associated with their organs. There were few and minimal α-TEA-related histomorphologic or organ weight changes observed at the end of dose administration in the adrenal glands (1500 mg/kg females), duodenum (1500 mg/kg males), jejunum (100 and 1500 mg/kg males and 1500 mg/kg females), kidney, liver (1500 mg/kg, male), and thymus (300 and 1500 mg/kg males and 100 and 1500 mg/kg females). These findings were considered to be incidental. No α-TEA related macroscopic changes were observed at the end of dose administration or recovery phase, and there were no changes in organ weight. Interestingly, α-TEA-related histomorphologic changes were noted at 1500 mg/kg on Day 29 in the duodenum (mild, diffuse villous atrophy) at 1500 mg/kg, jejunum (minimal, focal or multifocal dilation of a crypt) at all dose levels, kidney (mild, diffuse, bilateral vacuolation of the epithelium lining the proximal tubules) at 1500 mg/kg, and liver (minimal, multifocal hepatocellular necrosis with minimal, multifocal sub-acute inflammation and moderate, diffuse vacuolation of hepatocytes) at 1500 mg/kg. At the end of recovery period, minimal focal dilation of jejunal crypt and moderate diffuse vacuolation of hepatocytes was present at 1500 mg/kg and mild diffuse depletion of lymphocytes of the thymic cortex was noted at 300 mg/kg.

#### Toxicokinetics parameters

**Fig. 5:**
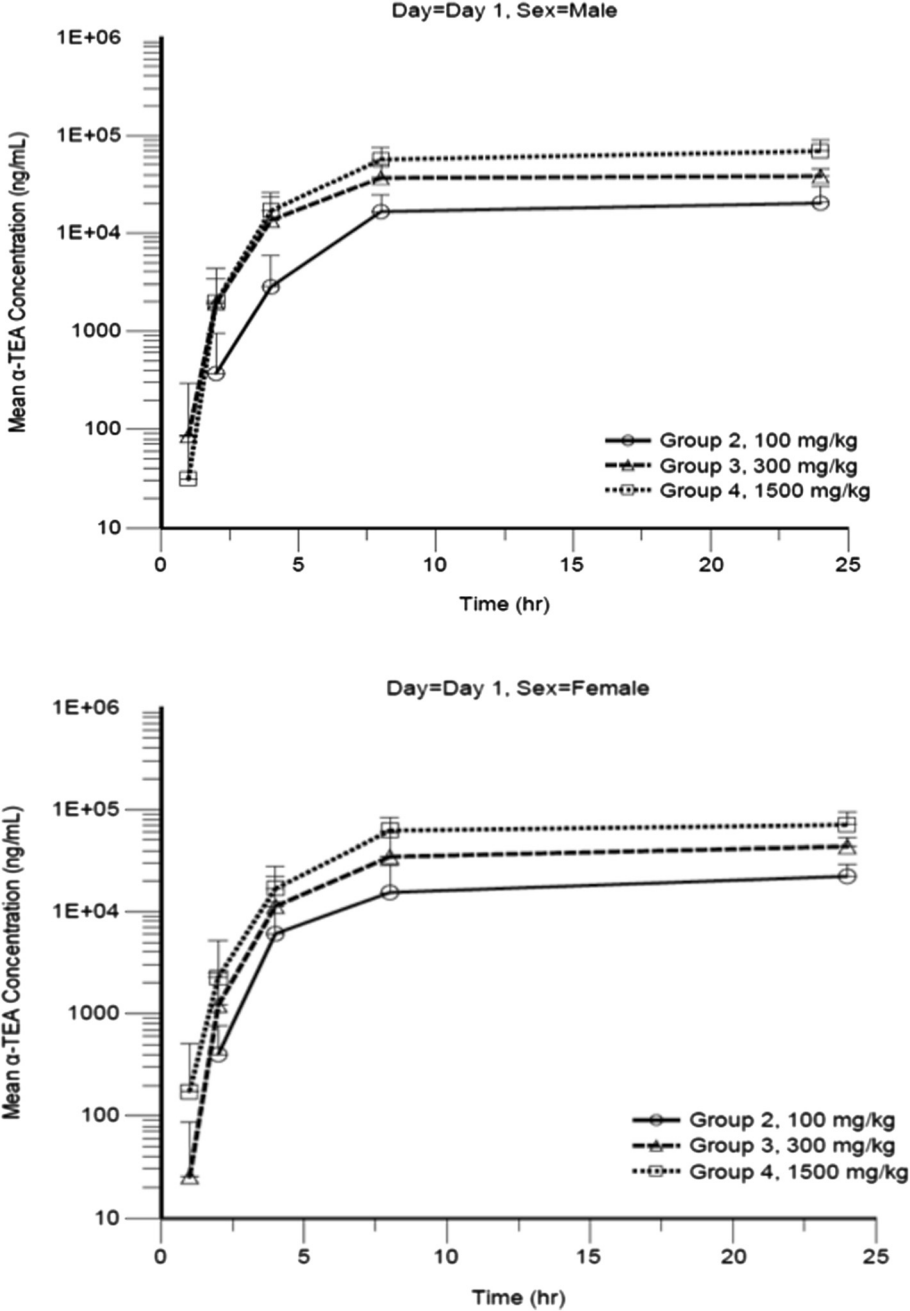
Mean plasma α-TEA lysine salt concentration versus time at day 1 for male and female dogs. Male dogs received indicated doses of α-TEA lysine salt on day 1. Plots of α-TEA plasma concentration versus time are presented

**Fig. 6:**
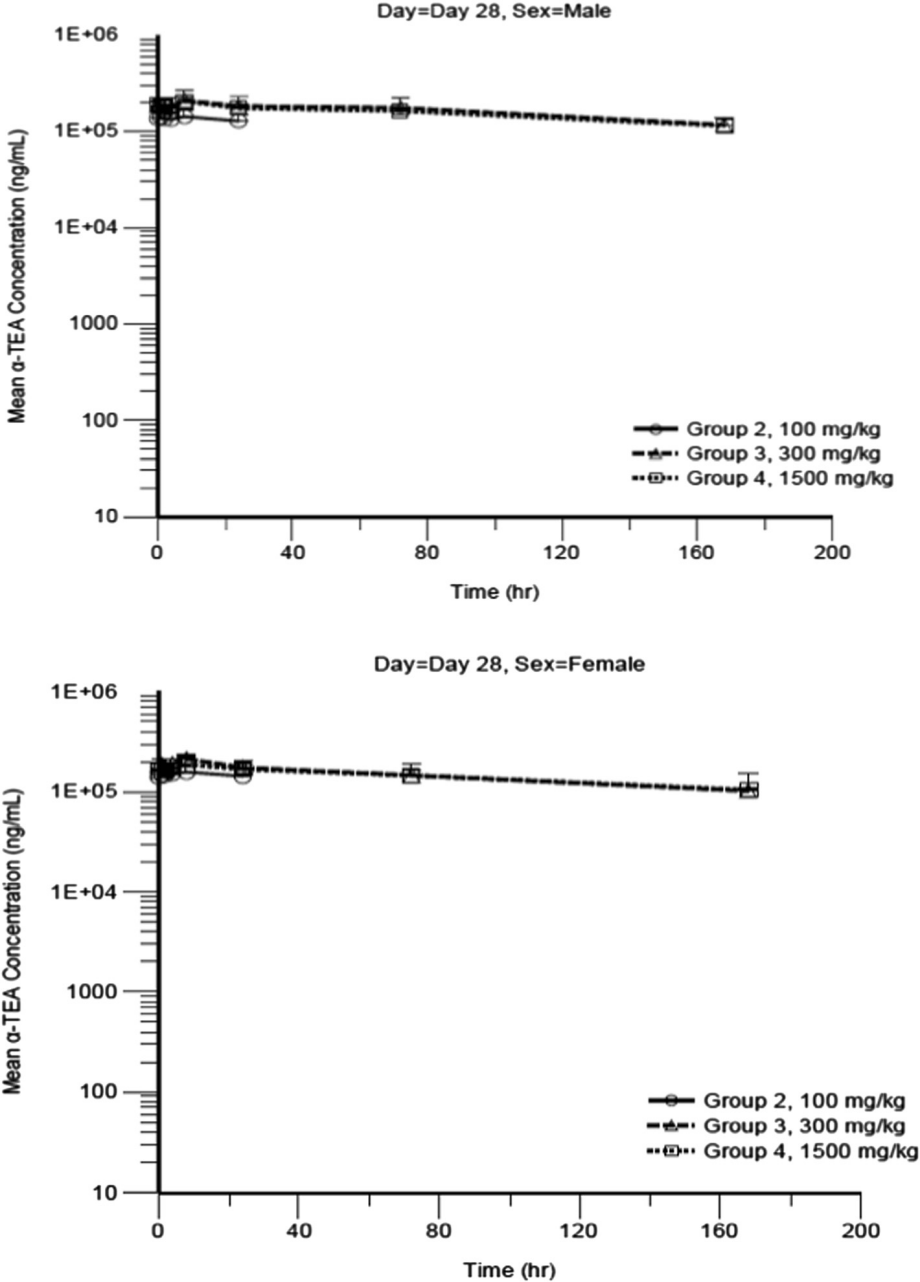
Mean plasma α-TEA lysine salt concentration versus time at day 28 for male and female dogs. Female dogs received indicated doses of α-TEA lysine salt on day 1. Plots of α-TEA plasma concentration versus time are presented

Samples of blood were collected from the jugular vein of all animals at 1, 2, 4, 8, 24 h at day 1 and day 28 when animals received the test article. The validated LC-MS/MS method was used to analyze plasma α-TEA concentration. Mean plasma α-TEA concentrations in all samples obtained at 8 h post-dose from control animals on Day 1 were below the lower limit of quantitation (50.0 ng/mL), indicating no exposure of treated animals to α-TEA prior to dosing. As shown in Table 9, the mean T_max_ ranged from 16.0 to 24.0 h on Day 1 and from 6.3 to 10.7 h on Day 28. Elimination half-life values were not calculated or not reported on Days 1 and 28 due to insufficient data points for the elimination phase. On Days 1 and 28, increases in C_max_ and AUC_0-24_ were less than proportional for both males and females. On the first day of administration, for a 15-fold increase in dose from 100 to 1500 mg/kg, C_max_ values increased 3.42-fold for males and 3.22-fold for females and AUC_0-24_ values increased 3.49-fold for males and 3.53-fold for females. On Day 28, α-TEA exposure was similar at 300 and 1500 mg/kg, this reflects a saturation phenomenon at 300 mg/kg dose level and above. For a 3-fold increase in dose from 100 to 300 mg/kg, C_max_ increased 1.43-fold for males and 1.33-fold for females and AUC_0-24_ increased 1.44-fold for males and 1.29-fold for females. C_max_ and AUC_0-24_ values were similar between males and females. The ratio of C_max_ for males to C_max_ for females at each dose level ranged from 0.84 to 0.90 on Day 1 and from 0.93 to 1.07 on Day 28. The ratio of AUC_0-24_ for males to AUC_0-24_ for females at each dose level ranged from 0.94 to 0.98 on Day 1 and from 0.91 to 1.09 on Day 28. The AUC ratios for α-TEA LS (ratio of AUC_0-24_ on Day 28 to the AUC_0-24_ on Day 1) was 9.82 for males and 10.31 for females at 100 mg/kg), 6.60 for males and 6.41 for females at 300 mg/kg, and 3.91 for males and 3.40 for females at 1500 mg/kg. Plots of α-TEA plasma concentration versus time are presented in Figs. 5 and 6 for Day 1 and Day 28, respectively. Exposure of animals on Day 28 was higher than exposure on Day 1, indicating accumulation of α-TEA with multiple dosing. The extent of accumulation was greatest at the low-dose level and decreased with increasing dose, presumably due to saturation of exposure at the mid- and high-dose levels. No sex-specific differences in toxicokinetics values could be reported.

**Table 9:** Mean toxicokinetic parameters at day 1 and 28-day dosing of α-TEA lysine salt

Day	Group	α-TEA Dose (mg/kg)	Sex	Tmax (hr)	T1/2 (hr)	Cmax (ng/mL)	AUC0-24 (hr*ng/ml)
1	2	100	Male	24	NC	20200	335000
1	2	100	Female	20	NC	23900	35100
1	3	300	Male	16	NC	41000	718000
1	3	300	Female	18.7	NC	48600	72900
1	4	1500	Male	24	NC	69100	1170000
1	4	1500	Female	16	NC	76900	1240000
28	2	100	Male	6.3	NC	148000	3290000
28	2	100	Female	7	NC	16000	3620000
28	3	300	Male	7.3	NC	211000	4740000
28	3	300	Female	8	NC	212000	4670000
29	4	1500	Male	8	NC	206000	4580000
28	4	1500	Female	10.7	NC	192000	4210000

Blood were collected collected at 1, 2, 4, 8, and 24 h following the first α-TEA administration and 2, 8, 24, 72, and 168 h following the last administration (day 28). The validated method (high performance liquid chromatography with mass spectrometric detection) was used to analyse plasma α-TEA concentration. C_max_, T_max_, T_1/2_, AUC were determined using WinNonlin Version 6.2 software system

## Discussion

This toxicology and pharmacokinetics study of GMP grade α-TEA was conducted to support an Investigator New Drug (IND) application to the FDA to conduct a first-in-human clinical trial in patients with advanced cancer based on the immunological and pro-apoptotic properties of α-TEA. Our results demonstrate that orally administered α-TEA LS reduces tumor growth and increases overall survival in mice bearing established tumors. This anti-tumor activity is comparable to that of α-TEA free acid that we previously reported [11]. However, unlike α-TEA free acid, which is difficult to manufacture for human use due to its liquid crystal properties, α-TEA LS is crystalline in nature and can be delivered in aqueous form to animals. In the non-rodent dose escalation study, in which optimal conditions and timing of orally administered α-TEA LS were assessed, there were no signs of toxicity in dogs that received daily doses of up to 1500 mg/kg. No moribundity or mortality was observed at any of the dose levels tested throughout the treatment period. No gender-related differences were observed. Minor histomorphologic alterations were observed at the highest dose but these changes were mostly reversed at the end of the recovery period.

Clinical pathology changes were limited to increased white blood cell (lymphocyte and neutrophil) counts, and fibrinogen levels at 1500 mg/kg; these changes were likely related to mild inflammation and/or stress but all these findings were resolved by the end of recovery period. Although a full and complete toxicokinetic profile could not be determined from the study, there was an apparent less than dose proportional increase in AUC over the duration of the study. The extent of accumulation was greatest at the low-dose level and decreased with increasing dose, presumably due to saturation of exposure at the mid- and high-dose levels.

## Conclusion

In summary, in this comprehensive examination of the prolonged use of GMP-grade α-TEA LS in dogs, followed by a 28-day recovery period, we report absence of any dose limiting toxicity. Our findings suggest that α-TEA is safe and tolerated and determined that the highest non-severely toxic dose (HNSTD) to be 1500 mg/kg. These findings formed the basis for initiating a first-in-human clinical trial of α-TEA in patients with advanced cancer, which is ongoing.

## Abbreviations

α-TEA: Alpha-Tocopheryloxyacetic free acid
α-TEA LS: α-TEA Lysine Salt
HPLC: High Performance Liquid Chromatography
